# Novel combination method of wide-range serial sectioning and 3D reconstruction visualizing both macro-level dynamics and micro-level interactions in an attempt to analyze the female pelvic floor

**DOI:** 10.1007/s12565-023-00710-0

**Published:** 2023-03-07

**Authors:** Satoru Muro, Keiichi Akita

**Affiliations:** Department of Clinical Anatomy, Tokyo Medical and Dental University, 1-5-45 Yushima, Bunkyo-ku, Tokyo, 113-8510 Japan

**Keywords:** 3D reconstruction, Anatomy, Histology, Methods, Pelvic floor

## Abstract

The present report presents details of the method for combining wide-range serial sectioning and 3D reconstruction using an adult cadaver. For several decades, anatomists have utilized a variety of non-destructive three-dimensional (3D) visualization methods to complement gross anatomical analysis methods. These include vascular casting for the visualization of vascular morphology and micro-CT for the visualization of bone morphology. However, these conventional methods are restricted by the properties and sizes of the target structures. Here, we introduce a method to conduct 3D reconstruction based on wide-range serial histological sections from adult cadavers, which overcomes previous restrictions. An attempt at 3D visualization of the female pelvic floor muscles provides a detailed description of the procedure. Supplemental video and 3D PDF files allow multifaceted observation of 3D images. Wide-range serial sectioning visualizes morphology beyond the scope of conventional methods, while 3D reconstruction enables non-destructive 3D visualization of any structure that can be observed on a histological section, including skeletal muscle, smooth muscle, ligaments, cartilage, connective tissue, blood vessels, nerves, lymph nodes, and glands. The novel combination of both methods is instrumental in meso-anatomy, a discipline intermediate between macro-anatomy and micro-anatomy.

**Supplementary Information:**

The online version contains supplementary material available at 10.1007/s12565-023-00710-0.

## Introduction

Anatomy focuses on the “form” as an object of observation. However, it is difficult to visualize precise structures while maintaining their three-dimensional (3D) structure using macroscopic anatomical methods (Muro et al. 2014, 2018, 2020, 2021a, c). In response to such challenges, anatomists have devised a variety of non-destructive methods for 3D visualization. For example, 3D imaging using micro-CT is a useful method for visualizing the 3D structure of bone (Clark and Badea 2021; Ritman 2011; Tharnmanularp et al. 2022; Tsukada et al. 2014; Tsutsumi et al. 2021). Using the difference in radiation absorbance of the tissue, the form is converted into digital data, and a 3D image is constructed using the software. 3D imaging using micro-CT has been widely applied to the study of clinical anatomy of the musculoskeletal system (Fujishiro et al. 2017; Fukai et al. 2022; Horiuchi et al. 2020; Hoshika et al. 2019; Momma et al. 2018; Nonthasaen et al. 2018; Nozaki et al. 2015; Saka et al. 2021; Sakaguchi-Kuma et al. 2016; Sato et al. 2018; Tamaki et al. 2014; Tano et al. 2021; Tsutsumi et al. 2019a, 2019b, 2020, 2022; Ueda et al. 2021). Although this method is excellent for non-destructive observation of bone morphology, it poorly depicts soft tissues, such as muscles and ligaments. The method that overcomes such limitations and allows 3D visualization of any tissue is the 3D reconstruction of histology.

3D reconstruction is a relatively well-established technique; however, recent developments in computers and software have made it possible to easily handle high volumes of 3D data. Previously, 3D reconstruction was performed on small samples, such as mice and human fetuses (Nyangoh Timoh et al. 2008; Nyangoh Timoh et al. 2018, 2020). This is due to size limitations in the preparation of serial histological sections. However, there are limitations to applying the anatomical findings of mice and human fetuses to adult anatomy and function. Furthermore, there is a large gap in the application of these findings in the understanding of adult pathology, diagnostic imaging, and surgery. Therefore, we developed a method to create a 3D reconstruction based on large serial sections of histology from adult cadavers; here it is called “wide-range serial sectioning”.

This paper describes a method for combining wide-range serial sectioning and 3D reconstruction using an adult cadaver through an attempt at 3D visualization of the female pelvic floor muscles near the urethra, vagina, rectum, and anal canal. The objective is to share the details of this method and demonstrate the 3D visualization of soft tissues.

## How to do wide-range serial sectioning and 3D reconstruction

**Fig. 1:**
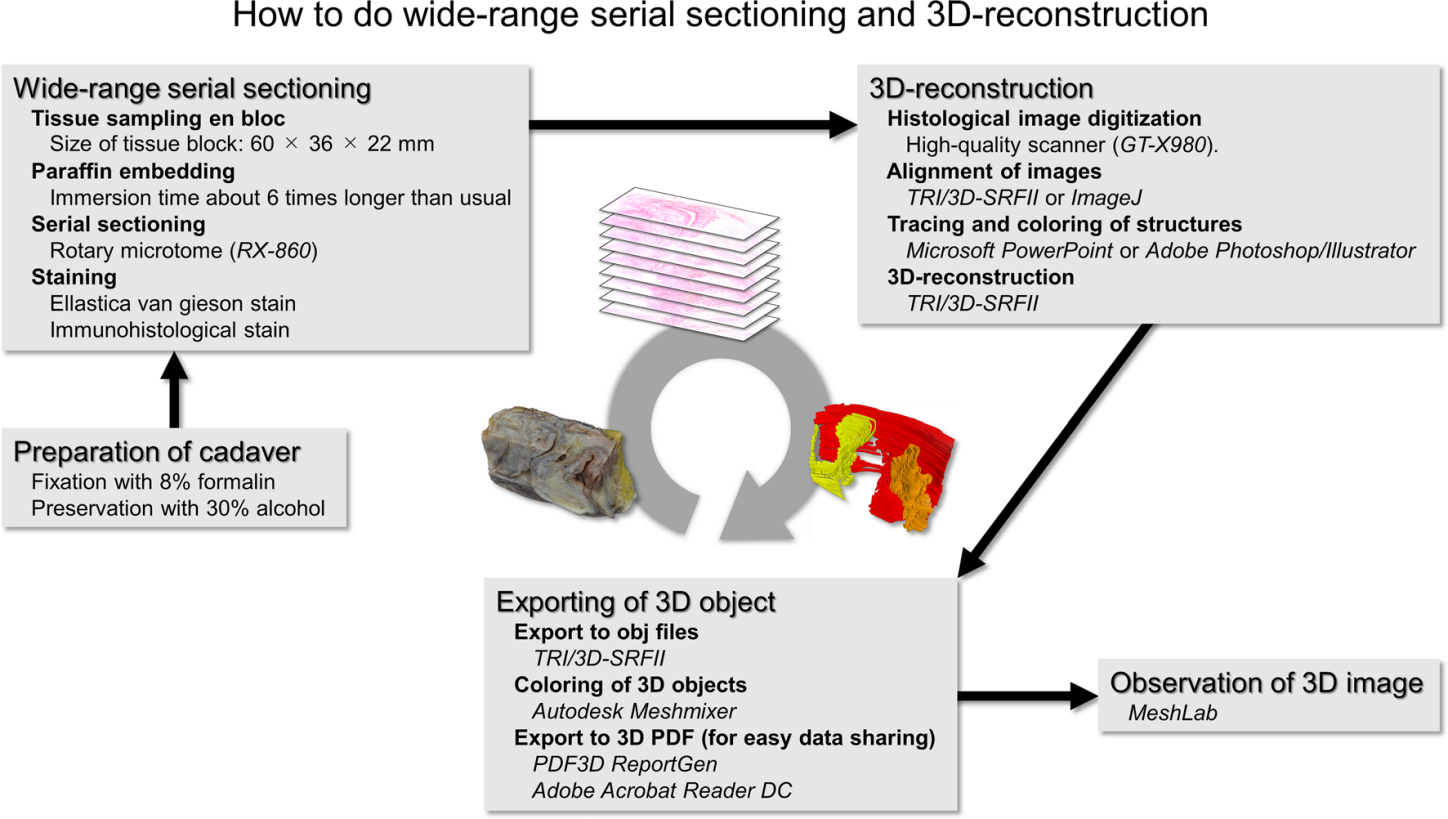
How to do wide-range serial sectioning and 3D reconstruction. The whole process of this new technique is depicted in the figure. Wide-range serial sectioning is characterized by a prolonged embedding duration due to the large size of the tissue block. Combining immunostaining is also advantageous. In 3D reconstruction, any structure that can be identified on histological sections can be transformed into a 3D object

Here, we describe the methods of wide-range serial sectioning and 3D reconstruction in detail (Fig. 1). As an example, we attempted a 3D visualization of the muscles using a female pelvic floor specimen.

### Preparation of cadaver

One female cadaver (83 years old at death) donated to our department was used in this study. The donation document format was congruent with the Japanese law entitled ‘The Act on Body Donation for Medical and Dental Education’ (Act No. 56 of 1983). Before their death, all donors voluntarily declared that their remains would be donated as material for education and study. At that time, the purpose and methods of using body donor corpses were explained and informed consent was obtained. After death, we explained the informed consent to the bereaved families and confirmed no opposition. All cadavers were fixed by arterial perfusion with 8% formalin and preserved in 30% alcohol. The study was approved by the Board of Ethics at Tokyo Medical and Dental University (approval number: M2018-006). All methods were performed in accordance with relevant guidelines and regulations.

The cadaver used in this examination was of an elderly individual with the age of 83, which may serve as a limitation to the study's outcomes. Given that older cadavers may possess more atrophied muscle fibers in comparison to younger cadavers, it is important to consider the use of younger cadavers in discussing normal structures. Nevertheless, no conclusive data indicate that the location of muscle attachments changes with age. Since the present study used cadavers without any history of surgery or disease in the pelvic region, it is believed that the assessment of the relative positioning of muscles and other structures should not be impeded.

### Wide-range serial sectioning

**Fig. 2:**
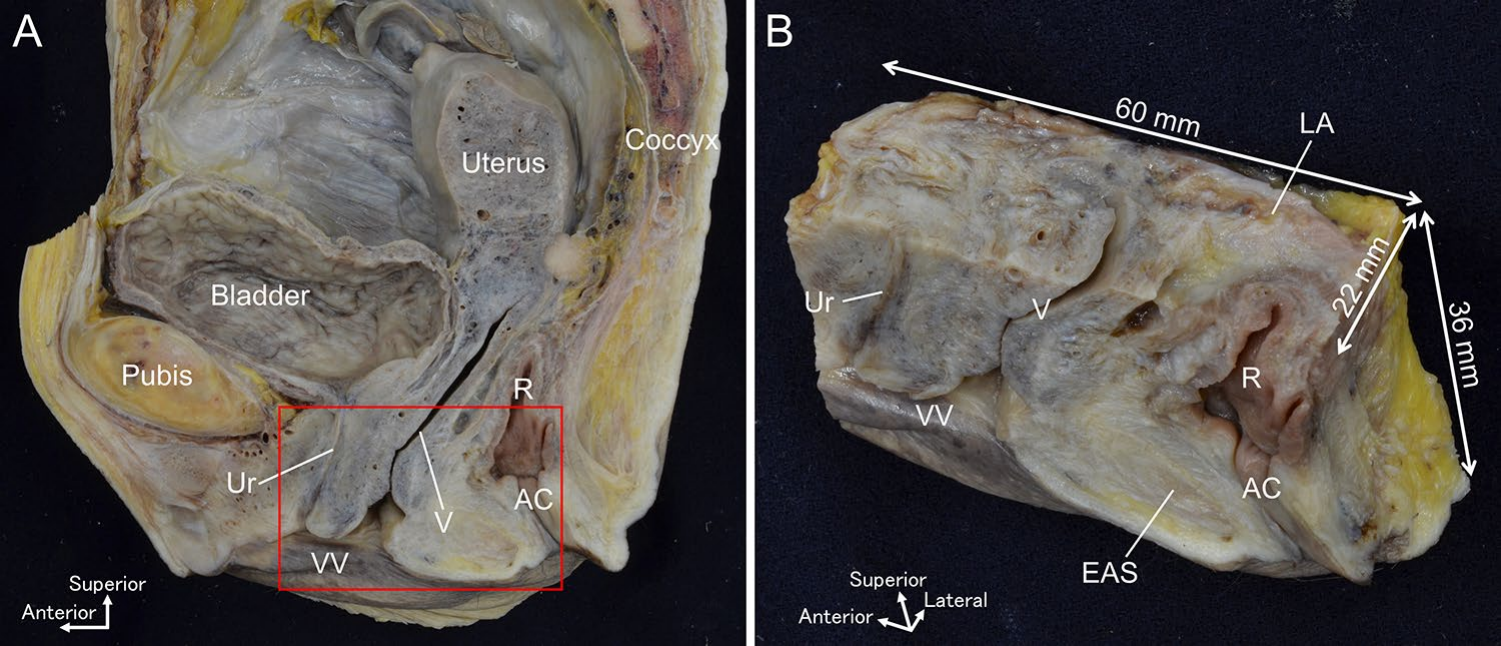
Collection of the wide-range tissue specimen. **A** Median section of the female pelvis. A tissue sample was obtained en bloc from the red rectangular area. **B** Wide-range tissue specimen obtained from the red rectangular area in (**A**). It includes almost the entire length of the urethra, the lower part of the vagina, the lower rectum and anal canal, and the levator ani near the viscera, 60 mm in diameter anteriorly, 36 mm in diameter vertically, and 22 mm in diameter laterally. AC, anal canal; EAS, external anal sphincter; *LA* levator ani; **R** rectum; *Ur* urethra; *V* vaginal, *VV* vaginal vestibule

The pelvis was obtained from the cadaver and sectioned in the median plane using a diamond band pathology saw (EXAKT 312; EXKAKT Advanced Technologies GmbH, Norderstedt, Germany) (Fig. 2A). Soft tissue of the pelvic floor was harvested en bloc (Fig. 2B). Incisions were made from the posterior margin of the pubic bone anteriorly to the posterior wall of the anal canal posteriorly, 60 mm in diameter anteroposteriorly, from the middle of the vagina superiorly and the skin of the perineum inferiorly, 36 mm in diameter vertically, and 22 mm in diameter laterally. The tissue block included almost the entire length of the urethra, lower part of the vagina, lower rectum and anal canal, and levator ani near the viscera. The size of such tissues is so large that they are unthinkable in normal histology. We refer to histological examination using such large tissue masses as “wide-range serial sectioning”.

In wide-range serial sectioning, the specimens are quite large and require significantly longer time than usual for embedding. The tissue blocks were fixed by immersion in 10% formalin for 24 h. The block was subsequently decalcified in Plank–Rychlo solution (AlCl3:6H2O 126.7 g/L, HCl 85 mL/L, HCOOH 50 mL/L) for 5 days. In elderly cadavers, decalcification is frequently necessary due to the calcification of vessels and organs. Following decalcification, neutralization was performed in a 5% sodium sulfate for 12 h. Thereafter, the tissue block was dehydrated (70% ethanol, 80% ethanol, 90% ethanol, 100% ethanol twice, xylene twice) for at least 24 h in the solution at each step. These immersion and fixation processes took approximately six times longer than usual, given the size of the tissue blocks. Thereafter, the block was embedded in paraffin for 5 days while applying negative pressure. The paraffin solution was changed thrice. A handmade container made from a milk carton was used to hold the 60 × 36 × 22 mm specimen, into which the specimen was placed, poured into paraffin, and hardened. Paraffin-embedded tissue blocks were serially sectioned in the transverse plane into 5-μm-thick specimens at 1-mm intervals using a rotary microtome (RX-860, Yamato Kohki Industrial Co. Ltd., Saitama, Japan).

Histological sections were stained with Elastica van Gieson. In addition, immunohistological staining of the sections was performed to confirm the distribution in skeletal muscle tissue. The slides were microwaved in 10 mM sodium citrate buffer (pH 6.0) for antigen retrieval. Endogenous peroxidase activity was inactivated by incubating tissues in methanol containing 0.3% H_2_O_2_ for 30 min. Nonspecific binding was blocked by incubation with phosphate-buffered saline containing 0.05% Tween 20 and 2.5% goat serum at room temperature for 30 min. The sections were incubated with primary antibodies against skeletal myosin (1:200, NBP-1–89,707, Myosin Heavy Chain 3 Antibody, Polyclonal; Novus Biologicals, CO, USA) overnight at room temperature. The sections were washed and incubated with peroxidase-conjugated anti-rabbit IgG reagent (ready-to-use, MP-7451, ImmPRESS^®^ HRP Goat Anti-Rabbit IgG Polymer, Vector Laboratories, CA, USA) as a secondary antibody for 30 min at room temperature. Immunocomplexes were detected using 3, 3-diaminobenzidine (Fujifilm Wako Pure Chemical Corporation, Osaka, Japan) and counterstained with hematoxylin for 1 min.

### 3D reconstruction

The stained specimens were scanned as whole slides using a high-quality scanner (GT-X980; Seiko Epson Corp., Tokyo, Japan). Image files of the scanned histological sections were aligned using the alignment function of TRI/3D-SRFII (ver. R.11.00.00.0-H, Ratoc, Tokyo, Japan; http://www.ratoc.com/eng/). Another method for the alignment of sections is to use the automatic alignment function of ImageJ (version 1.52; National Institutes of Health, Bethesda, Maryland, USA, https://imagej.nih.gov/ij/) (Schneider et al. 2012).

**Fig. 3:**
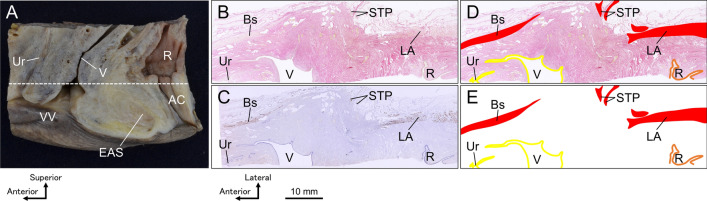
Structure identification, tracing, and coloring on histological sections. **A** Wide-range tissue block including the urethra, lower part of the vagina, lower rectum, and anal canal. The dotted line indicates the location of the transverse section from which the histological images in Figs. 3B and C were obtained. **B** Elastica van Gieson-stained image obtained from the transverse section indicated by the dotted line in (**A**). Pelvic viscera and surrounding skeletal muscles were observed. **C** Immunostaining images using anti-skeletal muscle antibodies. Bs, STP, and LA were stained as the skeletal muscles surrounding the pelvic viscera. STP displayed less vibrant color development in comparison to Bs and LA; however, the use of Elastica van Gieson staining (**B**) confirmed the presence of skeletal muscle fibers. **D** The skeletal muscles (Bs, STP, and LA) and the lumen of the urethra, vagina, rectum, and anal canal were identified, traced, and colored red, yellow, and orange, respectively. E: Image in which the histological section was removed (**D**). Image data such as this, in which the cross-sectional form of each structure is represented by color, are used for 3D reconstruction. *AC* anal canal; *Bs* bulbospongiosus; *EAS* external anal sphincter; *LA* levator ani; *R* rectum; *STP* superficial transverse perineal muscle; *Ur* urethra; *V* vagina; *VV* vaginal vestibule.

The following structures were traced and colored: skeletal muscles (the levator ani, external anal sphincter, bulbospongiosus, superficial transverse perineal muscle, and ischiocavernosus), the lumen of the urethra, vagina, rectum, and anal canal (Fig. 3). Tracing and coloring are most easily performed using the curve tool in Microsoft PowerPoint. Image processing software that can display layers, such as Adobe Photoshop or Illustrator, can also be used. Paste the image of the histological section and trace and color fill the structure on top in a separate layer.

**Fig. 4:**
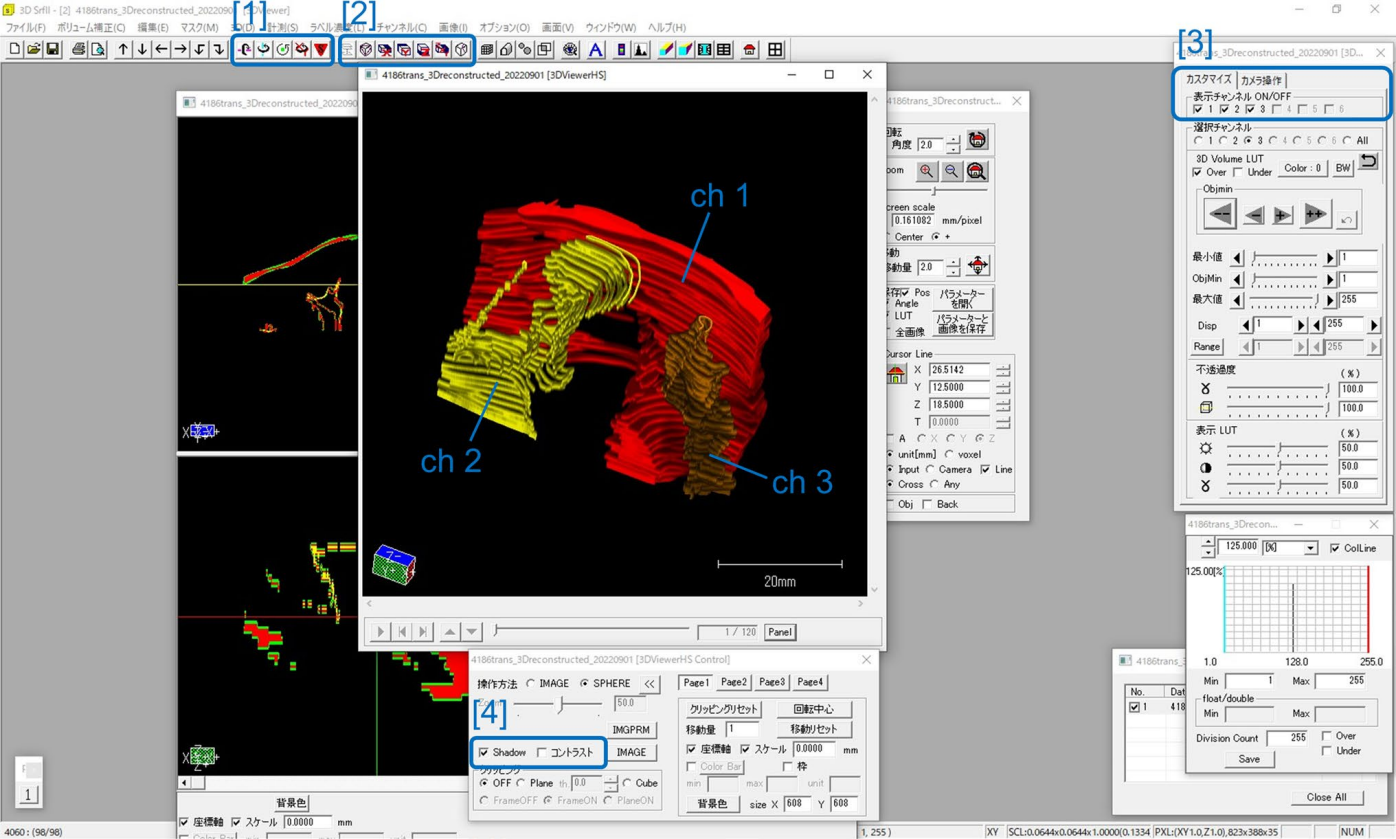
3D reconstruction using software. The section sequences were reconstructed using TRI/3D-SRFII. Skeletal muscles, represented in red, are stored in ch1; urethral and vaginal lumens, represented in yellow, are stored in ch2; and the lumen of the rectum and anal canal, represented in orange, are stored in ch3. The structure could be rotated by dragging the mouse. [1] are buttons to make movies of rotating the structure, [2] are buttons to make movies for cutting the structure, [3] are buttons to show or hide the structure in each channel, [4] are buttons to adjust shadow, contrast, light, etc. (see Supplementary Data 1 and 2)

The section sequences were reconstructed using TRI/3D-SRFII (Fig. 4). The following three steps were performed on the software: (1) extracting colored structures and storing them in each channel, (2) providing color information to each channel, and (3) volume correction (specify spacing and thickness). After reconstruction, a 3D image can be observed on TRI/3D-SRFII. The structure could be rotated by dragging the mouse. Movies for rotating the structure at various angles can be made ([1] in Fig. 4, Supplementary Data 1). Movies of the cutting cross sections of the structure at various angles can be made ([2] of Fig. 4, Supplementary Data 2). These movies could be exported as mpg files. The structures saved in each channel can be shown or hidden ([3] in Fig. 4). Parameters such as the display shadow, contrast, and light can be adjusted ([4] in Fig. 4).

### Exporting of 3D object

Using the obj export function of TRI/3D-SRFII, the structures saved in each channel were exported as obj files. The obj files exporting each structure were colored using Autodesk Meshmixer (ver. 3.1, Autodesk, CA, USA, https://www.meshmixer.com/). Skeletal muscles were red, vagina and urethra yellow, and rectum and anal canal orange.

Because TRI/3D-SRFII is dedicated and paid software, data reconstructed with TRI/3D-SRFII can only be opened on a PC with this software installed. If the data are exported to obj using the obj export function of TRI/3D-SRFII, the file can be opened using free viewer software. However, color information is lost with obj exports. Autodesk Meshmixer can be used to colorize obj files. If the file is heavy, mesh reduction can be performed using Autodesk Meshmixer. If there are any missing areas or unevenness due to misalignment or errors in slicing, smoothing and hole filling can also be performed in the Autodesk Meshmixer (not done in this case). It is also possible to combine separate obj files and export them into a single obj file.

The obj files were converted to a 3D PDF using PDF3D ReportGen (ver. 2.22.1.11324, Visual Technology Services Ltd., Berkshire, U.K., https://www.pdf3d.com/; VTS software, Tokyo, Japan, https://vts-software.co.jp/) (Supplementary Data 3). Obj files are relatively common file formats for 3D objects, but they require the installation of viewer software for 3D objects. In contrast, 3D PDF files can be opened using Adobe Acrobat Reader DC, a popular free software for PDF viewing. This is useful for sharing data with collaborators and can also be attached to papers, as in this study (Supplementary Data 3).

### Observation of 3D image

Obj files were observed using MeshLab (ver. 2022.02, ISTI-CNR, Rome, Italy, https://www.meshlab.net/) (Cignoni et al. 2008). MeshLab is a free 3D file viewer that can simultaneously open multiple obj files. We can rotate the structures by dragging with a mouse and show/hide each structure.

**Fig. 5:**
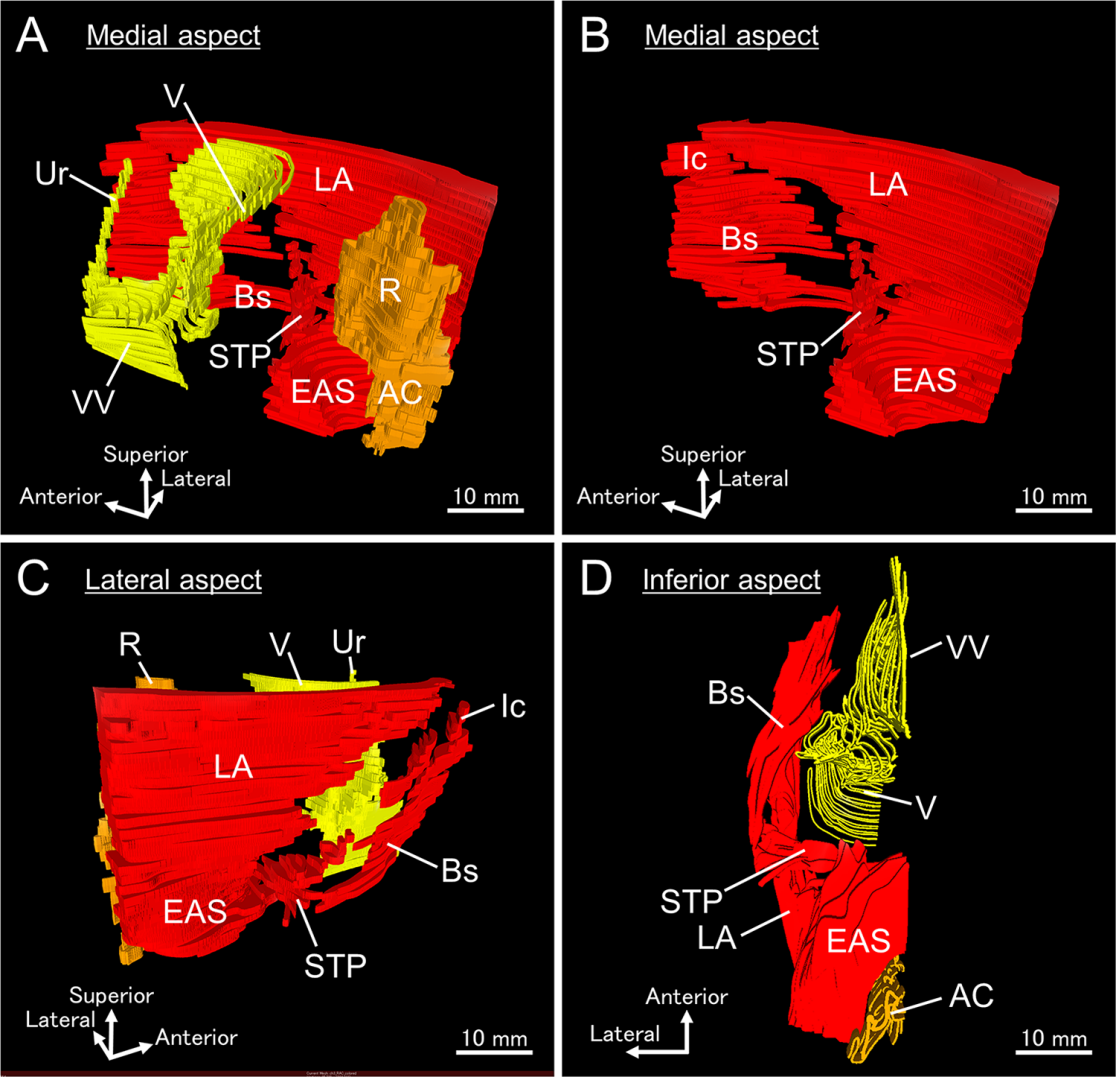
3D reconstructed image of female pelvic floor muscles. The three-dimensional arrangement of the pelvic floor muscles, including their spatial relationship with the pelvic viscera, was visualized. **A** Medial aspect showing the pelvic floor muscles (red), Ur and V (yellow), R and AC (orange). The LA and Bs ran laterally to the pelvic viscera. EAS surrounded AC. **B** Medial aspect showing only pelvic floor muscles, including the LA, EAS, Ic, Bs, and STP. LA and EAS were continuous. Bs runs posteriorly to the lateral side of the EAS. **C** Lateral aspect. STP medially adjoined to EAS on the anterior wall of AC. **D** Inferior aspect. Bs was located more superficially (inferiorly) than STP and ran toward the lateral side of the EAS. The STP was located more deeply (superiorly) than the Bs and adjoined the EAS on the anterior wall of the AC. *AC* anal canal; *Bs* bulbospongiosus; *EAS* external anal sphincter; *Ic* ischiocavernosus; *LA* levator ani; *R* rectum; *STP* superficial transverse perineal muscle; *Ur* urethra; *V* vagina; *VV* vaginal vestibule

As a result, the positional relationship between the pelvic floor muscles (skeletal muscles) and pelvic viscera (the urethra, vagina, rectum, and anal canal) was visualized in three dimensions (Fig. 5A). The levator ani and external anal sphincter surrounded the rectum and anal canal as continuous skeletal muscle structures (Fig. 5B). The innermost portion of the levator ani ran just lateral to the urethra and the vagina. The bulbospongiosus was located inferior to it, running posteriorly to the lateral side of the external anal sphincter. The superficial transverse perineal muscle ran from the lateral to the medial side, entering the confluence of the levator ani and external anal sphincter, and adjoining the external anal sphincter on the anterior wall of the anal canal (Fig. 5C, D). Regarding the superficial–deep relationship between the bulbospongiosus and superficial transverse perineal muscle when viewed from inferior aspect, the bulbospongiosus ran more superficially (inferiorly) toward the lateral side of the external anal sphincter, while the superficial transverse perineal muscle ran more deeply (superiorly) and adjoined to the external anal sphincter on the anterior wall of the anal canal (Fig. 5D). An important aspect of the present study is the discovery that the pelvic floor muscles are continuous with each other, without distinct boundaries. The name of each specific muscle referred to a segment of a continuous muscle complex. These findings are consistent with those presented by Baramee et al. and Suriyut et al. in their macroscopic examinations (Baramee et al. 2020; Suriyut et al. 2020), and we provided immunohistological support for such findings. The continuity of the pelvic floor muscles with one another indicates that these muscle groups do not function independently but rather constitute a seamless sheet and operate as an integrated system. The 3D arrangement of the pelvic floor muscles, including the spatial relationship with the pelvic viscera, is important to clarify the mechanism of pelvic floor support and the pathogenesis of pelvic organ dysfunction. The combination of wide-range serial sectioning and 3D reconstruction using adult cadavers is useful for morphological analysis of the pelvic floor and other soft tissues of the human body.

## Comparison with other methods, strengths and weaknesses

Non-destructive 3D visualization methods in anatomy include vascular casting (corrosion casting) (Cornillie et al. 2019; Eishi et al. 2021; Grabherr et al. 2016; Grandis et al. 2021; Wang et al. 2010) and 3D imaging using micro-CT (Clark and Badea 2021; Ritman 2011; Tharnmanularp et al. 2022; Tsukada et al. 2014; Tsutsumi et al. 2021), each of which has its own strengths and weaknesses. Vascular casting is excellent for hollow structures such as blood vessels, and micro-CT is excellent for bone visualization. However, soft tissues, such as muscles, ligaments, nerves, and lymph nodes, are difficult to visualize using these methods. With 3D reconstruction, any structure observed in a histological section can be visualized non-destructively in three dimensions. For example, skeletal muscles, smooth muscles, ligaments, menisci, cartilage, connective tissue, blood vessels, nerves, lymph nodes, and glands can be visualized (Muro et al. 2019, 2021b, 2022; Nasu et al. 2020). In addition, immunostaining is useful for the identification of skeletal muscles, smooth muscles, and nerves.

Previously, 3D reconstruction was performed on small samples, such as mouse and human fetuses (Ishii et al. 2021; Nyangoh Timoh et al. 2018, 2020; Yamaguchi et al. 2008). However, there are limitations in applying the anatomical findings of mice and human fetuses to adult anatomy and function because the fine arrangement and local microrelationships of muscle, connective tissue, microvasculature, and nerves in human adults may not be observed in mice and human fetuses. Recently, 3D reconstruction using human body slice images from visible human projects has been widely used (White et al. 2019; Wu et al. 2018). However, human body slice images have limitations in depicting histological structures, that is, fine muscle bundles, microvessels, and nerves cannot be depicted. 3D reconstruction using MRI has similar limitations (Larson et al. 2010).

## Novel combination method of wide-range serial sectioning and 3D reconstruction

**Fig. 6:**
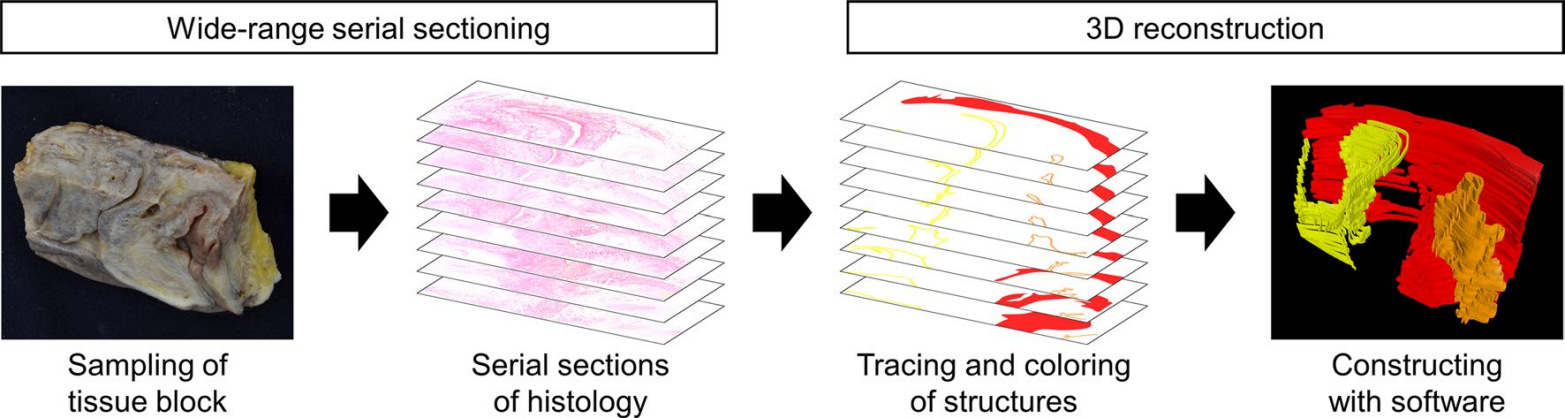
Novel combination of wide-range serial sectioning and 3D reconstruction. In wide-range serial sectioning, large tissue blocks are used to perform serial sectioning. This allows the search range to exceed that of the conventional ones. In 3D reconstruction, the structure is traced and colored, and the image data are reconstructed using software to obtain a three-dimensional image. 3D reconstruction enables non-destructive 3D imaging of any structure visible on histological sections. The novel combination of wide-range serial sectioning and 3D reconstruction is instrumental for meso-anatomy, a discipline between macro-anatomy and micro-anatomy

Our work of creating large serial histological sections from adult cadavers visualizes a range of morphology that is beyond the scope of previous methods of 3D reconstruction as described above (Muro et al. 2019, 2021b, 2022; Nasu et al. 2020). We call this “wide-range serial sectioning,” and the combination of wide-range serial sectioning and 3D reconstruction overcomes the difficulties of previous methods and allows us to capture the 3D extent of local histological structures in the human body (Fig. 6). Applications include the analysis of ligaments, menisci, cartilage, joint capsule, and surrounding muscles and tendons in the knee joint (Muro et al. 2022; Nasu et al. 2020), skeletal and smooth muscles around the rectum and anal canal (Muro et al. 2019), and the analysis of Cowper's glands and surrounding muscles in men (Muro et al. 2021b). Although unpublished, our research group has also applied this method to the analysis of fascia, blood vessels, nerves, and lymph nodes around the pancreas and stomach.

The size of tissue blocks handled in wide-range serial sectioning is so large that it is unbelievable in conventional histology. Therefore, long embedding times and sufficient sectioning techniques are required. However, sectioning and staining can be performed with an ordinary microtome and normal-sized glass slides, respectively, and installation of any special equipment is not required. It can be performed by a researcher or technician with basic sectioning skills and the time required for embedding is one to two months. Although a small number of stepwise serial sections (35 sections) were used in this study, finer 3D images can be obtained by acquiring a larger number of serial sections at shorter intervals. A beautiful 3D image was obtained using a series of 100–200 serial sections.

## Meso-anatomy as an intermediate research field

In recent years, clinical anatomy has been called upon to explore the 3D extent of histological structures. This is due to the remarkable development of endoscopic and robotic surgery and the increasing importance of quality of life (QOL) diseases. The new era of surgery, which has gained access and magnification capabilities, has allowed surgeons to encounter minute structures that are not covered by the conventional macroscopic anatomy. QOL diseases, such as dysphagia, pelvic floor dysfunction, and chronic pain, are not caused by a breakdown of macroscopic structures, such as fractures, bowel obstruction, myocardial infarction, and aortic dissection, but by a breakdown of smaller structures. These may be areas where the macroscopic anatomy cannot be reached. However, microscopic anatomy (histology) is more concerned with finer structures of cells and intracellular organs, and there is a dissociation from the needs of clinical anatomy. In other words, an intermediate level of analysis between macro- and micro-anatomy is required. This field of morphological research could be called “meso-anatomy”.

## Conclusions

This paper describes the methods for combining wide-range serial sectioning and 3D reconstruction using an adult cadaver. Wide-range serial sectioning visualizes morphology beyond the reach of conventional methods, while 3D reconstruction allows non-destructive 3D visualization of any structure that can be observed on a histological section, including skeletal muscle, smooth muscle, ligaments, menisci, cartilage, connective tissue, blood vessels, nerves, lymph nodes, and glands. The combination of wide-range serial sectioning and 3D reconstruction is expected to become a useful method in meso-anatomy, a discipline intermediate between macro-anatomy and micro-anatomy. Meso-anatomy works as an intermediary linking macro-level dynamics and micro-level interactions. The combination method of wide-range serial sectioning and 3D reconstruction described in this paper may be a useful method for meso-anatomy, visualizing the 3D extent of histological structures.

## Supplementary Information

Below is the link to the electronic supplementary material.

Supplementary file1 (MPG 1230 KB)

Supplementary file2 (MPG 482 KB)

Supplementary file3 (PDF 882 KB)

## Data Availability

All the relevant data used in this study can be accessed upon reasonable request from the corresponding author.
